# The role of transcription factor Yin Yang‐1 in colorectal cancer

**DOI:** 10.1002/cam4.5745

**Published:** 2023-03-06

**Authors:** Zhiying Shao, Wendong Yang, Xiannan Meng, Minle Li, Pingfu Hou, Zhongwei Li, Sufang Chu, Junnian Zheng, Jin Bai

**Affiliations:** ^1^ Cancer Institute, Xuzhou Medical University Xuzhou China; ^2^ Center of Clinical Oncology The Affiliated Hospital of Xuzhou Medical University Xuzhou China; ^3^ Jiangsu Center for the Collaboration and Innovation of Cancer Biotherapy Cancer Institute, Xuzhou Medical University Xuzhou China

**Keywords:** colorectal cancer, transcription factor, tumor promotion, tumor suppression, Yin Yang‐1

## Abstract

**Background:**

Yin Yang‐1 (YY1) is identified as a transcription factor with multiple functions. However, the role of YY1 in tumorigenesis remains controversial and its regulatory effects may depend upon not only cancer types, but also its interacting partners, chromatin structure, and the context in which it acts. It has been detected that YY1 was highly expressed in colorectal cancer (CRC). Intriguingly, many YY1‐repressed genes exhibit tumor suppressive potential while YY1 silencing is related to chemotherapy resistance. Therefore, it is crucial to meticulously explore YY1 protein structure and the dynamic alteration of its interactome in each cancer type. This review attempts to describe the structure of YY1, summarize the mechanism that influence the expression level of YY1 and also highlight the recent advances in our understanding of regulatory insights of YY1 functions in CRC.

**Methods:**

Related studies were identified through scoping search of PubMed, Web of science, Scopus and Emhase concerning the terms of “colorectal cancer”, colorectal carcinoma” or CRC with “YY1”. The retrieval strategy included title, abstract, and keywords with no language limitations. All the included articles were categorized depending on the mechanisms they explored.

**Results:**

In total, 170 articles were identified for further screening. After removing the duplication, not relevant outcomes and review articles, 34 were finally included in the review. Among them, 10 articles revealed the reasons of YY1 high expression in CRC, 13 articles explored YY1 function in CRC, and 11 articles fell into both aspects. In addition, we also summarized 10 clinical trials concerning the expression and activity of YY1 in various diseases, which offers a hint for future application.

**Conclusions:**

YY1 is highly expressed in CRC and broadly recognized as an oncogenic factor during the whole course of CRC. Sporadic controversial views are raised in term of CRC treatment, reminding us that future studies should take the influence of therapeutic regimens into concern.

## Graphical abstract

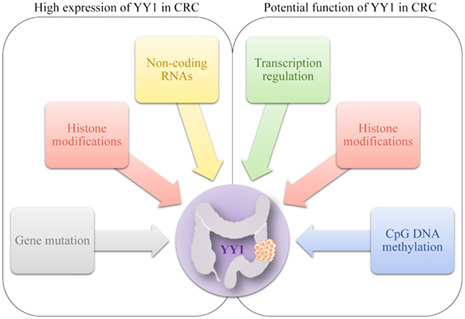

## 1 INTRODUCTION

YY1, also known as ∂ transcription factor, nuclear factor‐E1 (NF‐E1), INO80 complex subunit S and upstream control region binding protein, was first discovered in the early 1990s by several independent research groups.^1^, ^2^, ^3^, ^4^, ^5^ It was initially identified as a transcriptional repressor of adeno‐associated virus (AAV) P5 promoter. And then, its repression function was found to be reversed by adenovirus E1A oncoprotein and manifest as a transcription activator subsequently. Due to its dual transcriptional activity, the protein was named as Yin Yang‐1 (YY1) from the Chinese word “Yin” for repression and “Yang” for activation. Now, it is generally accepted that YY1 is ubiquitously expressed in human tissues to modulate the expression of various genes and cellular mechanisms including DNA repair, transcriptional regulation and epigenetic modifications. Meanwhile, YY1 may also be autoregulated via its own binding sites.^6^ The maintenance of proper YY1 expression level plays an important role in the normal function of cells and organisms.

Colorectal cancer (CRC) ranks third in incidence and second in mortality globally. International agency for research on cancer estimated more than 1.9 million new cases and 935,000 deaths in 2020, accounting for about one‐tenth of the total number of cancer cases and deaths.^7^ Patients who undergo radical CRC surgery at an early stage have a better prognosis, while patients with advanced‐stage diagnosis have a poor prognosis. With the breakthrough progress in CRC research, the median survival has remarkably improved in the past 20 years. However, there are still more than 50% of the patients suffering from metastatic disease at their first visits, which indicates a harsh survival.^8^ At the same time, with the continuous advancement of molecular biology technology in recent years, the regulation mechanism of malignant biological behavior of CRC has gradually become well‐defined, which is a complicated regulation process involving multiple genes and steps.^9^, ^10^

It has been detected that YY1 was highly expressed in a variety of cancer types including CRC. With the increase of differentiation grade of CRC, the expression level of YY1 increases, indicating a potential tumor promotion role of YY1 in CRC.^11^ Although many YY1‐repressed genes exhibit tumor suppressive potential, YY1 silencing may lead to chemotherapy resistance.^12^, ^13^ The role of YY1 in the promotion or suppression of tumor growth remains controversial and its regulatory effects may depend upon not only cancer types, but also its interacting partners, chromatin structure, and the context in which it acts at least in experimental settings.^14^ Therefore, it is crucial to meticulously delineate YY1 protein structure and the dynamic alteration of its protein–protein interactions (PPIs) in each cancer type, which may help to not only better understand its role in diverse cancer types but also design individual therapeutic strategies. This review describes the YY1 structure and summarizes the advances in our understanding of regulatory insights involving YY1 function in CRC.

## 2 YY1 SEQUENCE AND STRUCTURE

YY1 is a member of the GLI‐Kruppel family and polycomb protein family. Human YY1 gene, located in the telomere region q32.2 of chromosome No. 14, encodes a highly conserved protein of 414 amino acids with multiple functional domains including a transcriptional activation domain, a transcriptional repression domain and four C2H2‐type zinc fingers.^15^ The transcriptional activation domain (residues 1–69) is located in N‐terminal region of YY1 with acidic nature and involved in electrostatic interaction especially with positively charged proteins, while the transcriptional repression domain is located in the central region and the region near to C‐terminal of YY1. Four C2H2‐type zinc‐finger domains (residues 333–397) used to mediate DNA binding by interacting and recruiting various transcriptional regulators and proteins are located in the C‐terminal region of YY1.^15^, ^16^

Despite the multiple biological functions of YY1, its structure remains vague. The structures of three regions within YY1 are relatively well‐documented up to now. A comprehensive analysis using biochemical and biophysical techniques combined with bioinformatic approaches indicated that the N‐terminus of YY1 is a non‐compact fragment with little residual secondary structure but no well‐defined tertiary structure. The cocrystal structure of DNA‐binding domain (residues 295–414) in association with initiation element of AVV P5 promoter was obtained at 2.5‐Å resolution as a classic C2H2 zinc finger fold.^17^ It presents a series of features of disordered proteins when unbound, whereas a more stable conformation is established by interacting with DNA.^18^ All four fingers bind the major groove using the residues in the similar positions to those stabilizing the Zif268–, GLI–, and Tramtrack–DNA complexes.^19^, ^20^, ^21^ REPO domain named for its function in recruitment of Polycomb proteins was crystallized recently. Detected in a complex with the four human malignant brain tumor (4MBT) domain containing protein MBTD1, its crystal structure is uncovered as two anti‐parallel β strands connected by a short β hairpin loop.^22^ The short and highly conserved domain spanning amino acids 201–226 is necessary for Polycomb group recruitment to DNA, transcriptional repression and methylation of lysine 27 on histone H3.^23^ Taking all above into account, YY1 was considered to be the member of intrinsically disordered proteins (IDPs) with comparatively less globularity, low density, high flexibility, and dynamic behavior under physical conditions.^18^ While ensuring the multitasking ability of YY1, such disorder, however, hinders the observation of the full crystal structure of YY1.^24^ The well‐characterized functional domains of YY1 only partially overlap with the resolved structure mentioned above. YY1 structure using various experimental and bioinformatic means may provide significant path forward to its function and molecular mechanism.

## 3 HIGH EXPRESSION OF YY1 IN CRC

YY1 overexpression in CRC cells and tissues has been detected in previous studies, and the underlying mechanisms have also been explored partly (Table 1). Combining human genome database analysis and polymerase chain reaction (PCR)‐based single strand conformation polymorphism (SSCP) analysis, frameshift mutations of YY1 were detected in 4 human CRC tissues, all of which were subtypes with high microsatellite instability (MSI‐H). Among them, one case obtained one base deletion in the A8 repeat and the rest obtained one base duplication. However, the biological significance of such inactivating mutation was not elaborated further in this study.^25^ On the contrary, Chinnappan et al. found no evidence of gene amplification and chromosomal translocation but differential degree of aneuploidy and protein expression by comparing the multiple cancer cell lines or tissues with their normal counterparts, which implied the greater contribution of mRNA and protein stability to YY1 expression in CRC.^11^

**TABLE 1:** The potential mechanisms of YY1 high expression in CRC.

Cell lines	Xenograft	Patient samples	Mechanism	Reference
‐	‐	CRC tissues with MSI‐H or MSS	Frameshift mutations	^25^
LOVO, Caco‐2, HT‐29, and DLD‐1	‐	CRC tissues and normal colon tissues	mRNA and protein instability	^11^
‐	Nedd4‐knockout APC^min^ mice	‐	Nedd4, DN‐LEF1, Wnt	^26^
HT‐29, LOVO, SW480, HCT‐116, and human normal colonic epithelial cells (CCD‐18CO)	Nude mice with CRC cells injected subcutaneously	Paired CRC tissues and adjacent normal tissues	SMURF2, SENP1, c‐myc	^27^
HCT‐116 and 293 T	‐	‐	Feedback loop involving BCCIP	^28^
SW480, HCT‐116, LOVO, COLO205, HT‐29, CaCo‐2, and colonic epithelial cells (NCM‐460)	Athymic nude mice with CRC cells injected subcutaneously	Paired colon cancer tissues and adjacent normal tissues	OGT, SLC22A15, AANAT	^29^
‐	‐	CRC tissues and adjacent normal tissues	Hypomethylation	^30^
HCT‐116, DLD‐1, HT‐29, LOVO, SW480, and SW620	Female BALB/c nude mice with CRC cells injected subcutaneously	Paired CRC tissues and adjacent normal tissues	miR‐7, p53, Wnt	^31^
HCT‐116, DLD‐1, HT‐29, SW480, SW620, and colonic epithelial cells (NCM‐460)	‐	CRC tissues and control tissues from disease‐free donors	CRHR2/Ucn2, miR‐7, Fas/FasL	^32^
HCT‐116, HT‐29, SW480, SW620, DLD‐1, and human normal colon epithelial cells (FHC)	Male BALB/c nude mice with CRC cells injected subcutaneously	CRC tissues and adjacent normal tissues	c‐Myb, circHIPK3, miR‐7	^33^
SW480	‐	‐	miR‐34a, p53	^34^
SW480, HCT116, SW620, HT‐29, and human colon epithelial cells (NCM‐460)	Male BALB/c nude mice with CRC cells injected subcutaneously	CRC tissues and adjacent normal tissues	circAGFG1, miR‐4262, miR‐185‐5p, CTNNB1, Wnt	^35^
HT‐29, HCT‐116, LS174T, SW620, and LOVO	‐	Paired CRC tissues and adjacent normal tissues	miR‐186	^36^
LS174T, LOVO, HT‐29, HCT‐116, SW480, and SW620	‐	Paired CRC tissues and adjacent normal tissues	miR‐215	^37^
RKO, SW480, SW620, LOVO, HCT116, and human umbilical vein endothelial cells (HUVECs)	Nude mice with CRC cells injected subcutaneously for tumorigenicity assay and injected into tail vein for metastasis assay	Paired CRC tissues and adjacent normal tissues	MIR31HG, miR‐361‐3p	^38^
HT‐29, SW620, LOVO, HCT‐116, and the human colon epithelium cells (FHC)	Male BALB/c mice with CRC cells injected subcutaneously	Fresh CRC tissues and adjacent normal tissues	LINC00667, miR‑449b‑5p	^39^
‐	‐	Fresh CRC tissues and adjacent normal tissues	miR‐4728‐5p	^40^
HCT‐116 and CCL‐244	‐	CRC tissues and adjacent normal tissues	lnc‐TLCD2‐1, miR‐193a‐5p, NF‐кB, p65	^41^
LOVO, Caco‐2, SW620, HT29, HCT‐116, and normal colon epithelial cells (NCM‐460)	‐	Paired CRC tissues and adjacent normal tissues	NEAT1, miR‐216b	^42^
DLD‐1, LOVO, HCT‐116, HT‐29, and Caco‐2 and normal epithelial colon cells (NCM‐460)	Male BALB/c mice with CRC cells injected subcutaneously for tumorigenicity assay and with injected into the splenic artery for metastasis assay	Fresh CRC tissues and matched normal tissues	TCONS_00012883, DDX3, MMP1, PI3K/AKT	^43^
HCT‐116 and LOVO	BALB/c female nude mice with CRC^†^ cells injected subcutaneously	Fresh CRC tissues	Circ‐CTNNB1, DDX3	^44^

Abbreviations: CRC, colorectal cancer; MSI‐H, microsatellite instability‐high; MSS, microsatellite stability.

### 3.1 Histone modifications are responsible for YY1 overexpression in CRC

Epigenetic modifications refer to any reversible, heritable changes in gene expression without changes in the DNA sequence, including three main molecular events: CpG DNA methylation, non‐coding RNA regulation and various histone modifications. The last one contains all the histone modification process under the action of related enzymes, such as methylation, acetylation, phosphorylation, adenylation, ubiquitination, and ADP ribosylation.^45^, ^46^ An E3 ubiquitin ligase, neuronal precursor cell developmentally down‐regulated 4 (Nedd4) was reported to degrade YY1 via ubiquitination.^26^ In line with this, another E3 ubiquitin ligase, SMAD ubiquitination regulatory factor 2 (SMURF2) is weakly expressed in CRC tissues and cells and were also reported to ubiquitinate YY1.^27^, ^47^, ^48^ Interestingly, human BRCA2 and CDKN1A/p21‐interacting protein (BCCIP), an evolutionarily conserved nuclear protein, was detected to negatively regulate YY1 ubiquitination by binding directly to YY1 at the residues that was partially overlapped with the binding site of SMURF2. However, knocking down SMURF2 failed to reduce shBCCIP‐mediated increase of YY1‐Ubi, indicating the mutual independence of the two proteins.^28^

In contrast to ubiquitination, O‐GlcNAcylation by O‐GlcNAc transferase (OGT), On the other hand, enhances the stability of YY1, resulting in CRC progression by regulating multiple oncoproteins (e.g., SLC22A15 and AANAT).^29^ Through a methylated DNA immunoprecipitation‐chip analysis of a paired CRC and adjacent normal tissues, YY1 was also screened to be one of the prominent transcription factors (TFs) with aberrant promoter hypomethylation which might be responsible for its high expression level and tumorigenesis function.^30^

### 3.2 Non‐coding RNA regulation is responsible for YY1 overexpression in CRC

Besides histone modifications, YY1 can be targeted directly or indirectly by a variety of non‐coding RNAs (ncRNAs) that act as tumor suppressors. miRNAs are a group of ~22‐nucleotide endogenous small ncRNAs that silence gene expression at the post‐transcriptional level. By interacting with the 3′ untranslated region (3′ UTR) of mRNAs, miRNAs participate in the regulation of oncogenes and tumor suppressor gene expressions.^49^ Some miRNAs are known to be tissue‐specific and deregulated in CRC patients, such as let‐7, miR‐9, miR‐17, miR‐19, miR‐21, miR‐24, and miR‐155.^50^ Up to date, YY1 expression has been reported to be negatively regulated by miR‐7, miR‐34a, miR‐185‐5p, miR‐186, miR‐193a‐5p, miR‐215, miR‐361‐3p, miR‐449b‐5p, miR‐4262, and miR‐4728‐5p in CRC.^31^, ^32^, ^33^, ^34^, ^35^, ^36^, ^37^, ^38^, ^39^, ^40^

Long non‐coding RNAs (lncRNAs) are detected in various biological processes, including cell differentiation, proliferation, apoptosis, and migration. A body of evidence have proved the existence of dysregulated lncRNAs in the progression of different tumors.^51^ lncRNAs may also regulate YY1 activity, however, usually in an indirect mode by sponging miRNAs as competing endogenous RNAs (ceRNAs), such as lnc‐TLCD2‐1 and NEAT1.^41^, ^42^ Another study found that TCONS_00012883, a novel lncRNA, mediates the transactivation of YY1 by binding DEAD‐box helicase 3 (DDX3) protein to promote the interaction between DDX3 and YY1, thus resulting in transcription of MMP1 and activation of the PI3K/AKT pathway.^43^ Unfortunately, this study failed to identify the specific binding sites on DDX3 protein, which might be the key of targeted therapy. On the other hand, a recent study from Yang and colleagues confirmed that the Ia domain of DDX3 protein bind with circ‐CTNNB1 to increase its interaction with YY1, resulting in the transactivation of YY1. Accordingly, they designed a cell‐penetrating peptide, named DIP‐13 to target the binding domain and confirmed its suppression function to circ‐CTNNB1.^44^

Circular RNAs (circRNA), a group of highly conserved transcripts characterized by covalently closed continuous loops, are assembled with exons or introns of parent genes.^52^ CircRNAs are considered to be related with diverse human diseases, especially cancers.^53^, ^54^ Similar to lncRNAs, circ‐AGFG1 was found to sponge miR‐4262 and miR‐185‐5p, resulting in upregulation of YY1 expression.^35^ YY1 is also reported as one of the proto‐oncogenes regulated by c‐Myb/circ‐HIPK3/miR‐7 axis.^33^

## 4 REGULATING FUNCTIONS OF YY1 IN CRC

### 4.1 The transcriptional factor action of YY1

Universally, YY1 functions as a transcriptional factor by directly binding to the promoter regions of downstream genes to regulate their expression, and then exert oncogenic function via multiple canonical pathways (Table 2).

**TABLE 2:** The potential mechanisms of YY1 function in CRC.

Cell lines	Xenograft	Patient data	Mechanism	Outcomes	Reference
HT‐29, LOVO, SW480, HCT‐116, and human normal colon fibroblasts (CCD‐18Co)	Nude mice injected with CRC cells	Paired CRC tissues and adjacent normal tissues	SMURF2, SENP1, c‐myc	Promoting the malignant phenotypes and tumorigenicity	^27^
‐	Nedd4‐knockout APCmin mice	‐	Nedd4, DN‐LEF1, Wnt	Promoting tumor growth	^26^
SW480, HCT‐116, LOVO, COLO205, HT‐29, CaCo‐2, and human normal colon epithelium cells (NCM‐460)	Athymic nude mice with CRC cells injected subcutaneously	Paired colon cancer tissues and adjacent normal tissues	OGT, SLC22A15, AANAT	Stimulating tumorigenesis in CRC cells	^29^
HCT‐116, DLD‐1, HT‐29, LOVO, SW480, and SW620	Balb/c nude mice with CRC† cells injected subcutaneously	Paired colon cancer tissues and adjacent normal tissues	miR‐7, p53, Wnt	Inducing proliferation but reducing apoptosis of CRC; Indicating poor prognosis of CRC patients	^31^
HCT‐116, DLD‐1, HT‐29, SW480, SW620, and human normal colon epithelium cells (NCM‐460)	‐	CRC tissues and control tissues from disease‐free donors	CRHR2/Ucn2, miR‐7, Fas/FasL	Leading to tumor cell resistance of Fas/FasL‐apoptosis	^32^
SW480, HCT116, SW620, HT‐29, and human normal colon epithelium cells (NCM‐460)	Male BALB/c nude mice with CRC cells injected subcutaneously	CRC tissues and adjacent normal tissues	circAGFG1, miR‐4262, miR‐185‐5p, CTNNB1, Wnt	Promoting the metastasis and stemness in CRC	^35^
SW480, SW620, LOVO, HCT116, and DLD1	Nude mice with CRC cells injected subcutaneously	‐	TMEM97, β‐catenin	Promoting tumor growth but reducing apoptosis of CRC	^55^
HCT116, SW480, LOVO, SW116, and the human normal colon epithelium cells (FHC)	‐	CRC tissues	DDX11‐AS1, miR‐873, CLDN7	Promoting tumor progression	^37^
DLD‐1, LOVO, HCT‐116, HT‐29, and Caco‐2 and human normal colon epithelium cells (NCM‐460)	Male BALB/c mice with CRC cells injected subcutaneously for tumorigenicity assay and with injected into the splenic artery for metastasis assay	Fresh CRC tissues and matched normal tissues	TCONS_00012883, DDX3, MMP1, PI3K/AKT	Promoting proliferation and metastasis of CRC	^56^
SW620, SW480, HT‐29, LOVO, HCT‐116, and human normal colon epithelium cells (HCoEpiC)	‐	CRC tissues and adjacent normal tissues	ARAP1‐AS1, Wnt	Promoting tumor cell migration and invasion	^57^
SW620, LOVO, SW480, SW1116, HCT‐116, DLD‐1 Caco‐2, and human normal colon epithelium cells (FHC and NCM‐460)	Female BALB/c nude mice with CRC cells injected subcutaneously	CRC tissues and adjacent normal tissues	miR‐500a‐5p, HDAC2, p300	Promoting tumor cell proliferation and migration	^58^
HCT‐116, SW620, RKO, and human normal colon epithelium cells (HIEC)					
‐	CRC tissues and adjacent normal tissues	miR‐526b‐3p, HDACs	Promoting tumor cell proliferation; Indicating poor prognosis	^12^	
HCT‐116 and CCL‐244	‐	CRC tissues and adjacent normal tissues	lnc‐TLCD2‐1, miR‐193a‐5p, NF‐кB, p65		
	Inducing radiation resistance of CRC	^41^			
DLD‐1, HT‐29, LS174T, SW620, and human normal colon epithelium cells (HIEC‐6)	‐	‐	NF‐κB, EZH2, ST6GalNAc6, DTDST	Promoting colorectal carcinogenesis at early stage	^59^
‐	‐	‐	EZH2, VEGF	Involving in the interaction network between EZH2 and VEGF	^60^
‐	‐	DNA samples from CRC and normal colon	de novo methylated CpG islands	Maintaining the unmethylated status of CpG islands by cooperative binding with other transcription factors	^61^
RKO, SW480, SW620, LOVO, HCT‐116, and HUVECs	Nude mice with CRC cells injected subcutaneously for tumorigenicity assay and injected into tail vein for metastasis assay	Paired colon cancer tissues and adjacent normal tissues	MIR31HG, miR‐361‐3p	Promoting the proliferation and glycolysis of CRC cells as well as the angiogenesis of HUVECs^‡^	^38^
HT‐29, SW620, LOVO, HCT‐116, and human normal colon epithelium cells (FHC)	Male BALB/c mice with CRC cells injected subcutaneously	Fresh CRC tissues and adjacent normal tissues	Feedback loop involving LINC00667 and miR‑449b‑5p	Promoting CRC cell growth and migration	^39^
SW480, HCT‐116, SW620, LOVO, and human normal colon epithelium cells (FHC)	Male BALB/c athymic mice with CRC cells injected subcutaneously	CRC tissues and adjacent normal tissues	Feedback loop involving LINC01224, miR‐485‐5p and MYO6	Promoting CRC cell proliferation, migration, and invasion	^62^
SW480, HT‐29, LOVO, DLD‐1, HCT‐116, Caco‐2, and human normal colon epithelium cells (NCM‐460)	BALB/c male mice with CRC^†^ cells injected into the spleen for liver metastasis assay	Paired colon cancer tissues and adjacent normal tissues	Feedback loop involving LINC01578, NFKBIB and EZH2	Promoting colon cancer metastasis	^63^
HCT‐116 and 293 T	‐	‐	Feedback loop involving BCCIP	‐	^28^
HCT‐116, Caco‐2, HT‐29, and SW620	‐	‐	BCL2L15	Positively regulating 5‐Fluorouracil‐induced cytotoxicity	^13^
‐	‐	345 CRC patients	Loss of YY1 expression	Associated with aggressive phenotypes and poor patient outcome in AJCC stage III CRC.	^64^
DLD‐1 and SW48	‐	143 CRC patients	ITGAV, ITGB1	Acting as a tumor suppressor and related to better survival of patients with CRC	^65^

Abbreviations: AJCC, American joint Committee on cancer; CRC, colorectal cancer; HUVECs, human umbilical vein endothelial cells.

Degraded YY1 was observed to inactivate the SUMO specific peptidase 1 (SENP1)/MYC proto‐oncogene (c‐myc) axis, resulting in the suppression of malignant phenotypes and tumorigenicity of CRC cells.^27^ Several studies of the p53‐signaling pathway showed that YY1 could suppress the positive regulators (e.g., E2F1 and PARP) of the p53‐signaling pathway and promote downstream proto‐oncogene c‐Jun.^31^ Through a p53‐dependent manner, YY1 may also suppress apoptosis by upregulating anti‐apoptotic MCL1 while downregulating pro‐apoptotic caspase 3, caspase 7, caspase 9, and GML1. Meanwhile, YY1 might be a transcriptional repressor of Fas in CRC to suppress Fas/FasL‐mediated apoptosis, and the miR‐7/YY1/Fas cascade is mediated by corticotropin‐releasing hormone receptor 2 (CRHR2)/urocortin 2 (Ucn2) signaling.^32^

In adenomatous polyposis coli (APC)‐derived tumor models, overexpressed YY1 activates Wnt signaling, thus resulting in augmented tumor growth. Mechanistically, YY1 acts as a suppression factor of second promoter in intron 2 (P2) of lymphoid enhancer factor 1 (LEF‐1) to silence dominant negative LEF‐1 isoform, which allows full‐length LEF‐1 and other full‐length TCF family members to act unopposed in their regulation of Wnt target genes.^26^ Meanwhile, YY1 stimulates the Wnt‐signaling pathway by the downregulation of Wnt antagonists, including CSNK1A1, CTNNBIP1, SFRP1, and upregulation of Wnt‐initiating components (e.g., Wnt1, Wnt3a, Wnt4, FZD4, and β‐catenin).^31^, ^35^ YY1 can also indirectly modulate the Wnt/β‐catenin signaling pathway by transcriptionally activating transmembrane protein 97 (TMEM97), a conserved integral membrane protein that promotes β‐catenin stabilization.^55^

Under the regulation of ncRNAs, YY1 could also influence the function of ncRNAs in turn. Some microRNAs, such as miR‐500a‐5p and miR‐526b‐3p, have been detected to be silenced by YY1.^12^, ^58^ YY1 could function as a transcription activator by directly binding to lncRNAs. DDX11 antisense RNA 1 (DDX11‐AS1) was a novel CRC‐related lncRNA that highly expressed in CRC specimens and cell lines. With the screening of transcription factor databases, Chromatin immunoprecipitation and luciferase reporter assays, YY1 was proved to be a positive regulator of DDX11‐AS1 transcription by binding to its promoter regions. YY1‐mediated DDX11‐AS1 overexpression leads to competitive bind of miR‐873 and releases CLDN7 (Claudin 7) from miRNAs‐mediated degradations, thus facilitating the CRC progress.^56^ Similar interaction was also observed between YY1 and lncRNA ARAP1 antisense RNA 1 (ARAP1‐AS1).^57^

### 4.2 The epigenetic modifications involving YY1

In addition to directly binding to the promoter region of targeted genes, YY1 can also recruit other factors or complex to function together (Table 2).

Global evidence has shown that YY1 could interact with histone acetyltransferase p300 and histone deacetylases (HDACs) to form YY1/p300/HDAC2, YY1/p300/HDAC3, and YY1/p300/HDAC6 complexes.^58^, ^66^, ^67^, ^68^ In CRC, YY1 was detected to cooperate with HDAC2 to suppress miR‐500a‐5p promoter transcription, while p300 moderates the effect by binding to the upstream YY1‐binding sites of miR‐500a‐5p promoter in CRC cells.^58^ In line with this, Fang et al. found that YY1 failed to silence miR‐526b transcription in CRC cells with the treatment of HDAC inhibitor trichostatin A, indicating the co‐repression effect of YY1 and HDACs.^12^ The expression of YY1/nuclear factor‐κ‐gene binding P65 (NF‐кB‐P65) complex indirectly regulated by Lnc‐TLCD2‐1 may negatively mediate the radio‐sensitivity of CRC cell lines by regulating tumor microenvironment infiltration of immune cells.^41^

One study showed that YY1 is responsible for the epigenetic silencing of DTDST and ST6GalNAc6 (functioning to maintain the synthesis of normal glycans) in early‐stage CRC by recruiting histone repressive complex, polycomb repressive complex 2 (PRC2) containing enhancer of zeste homolog 2 (EZH2) to their promoter regions. The process is triggered by NF‐κB who positively mediate EZH2 transcription.^59^ The result is consistent with a recent study that confirms the strong link between YY1 and EZH2 via bioinformatic analysis.^60^

Despite the role of YY1 that positively regulating histone methylation, it is considered to prevent or at least impede de novo CpG DNA methylation, suggesting the interdependence and antagonism of two epigenetic modifications. An earlier study from Glaudia and colleagues provided both experimental and computational evidence that some general TFs including YY1 play a protective role in maintaining the unmethylated status of specific CpG islands of both normal and malignant cells. Meanwhile, it relies on the combinatorial binding of two or more TFs to provide such reliable protective chromatin environment.^61^ Further functional validation is required to prove the speculation that the aberrant methylation patterns in cancer cells partially arise from the loss of such protection.

### 4.3 Feedback loop involving YY1

**FIGURE 1:**
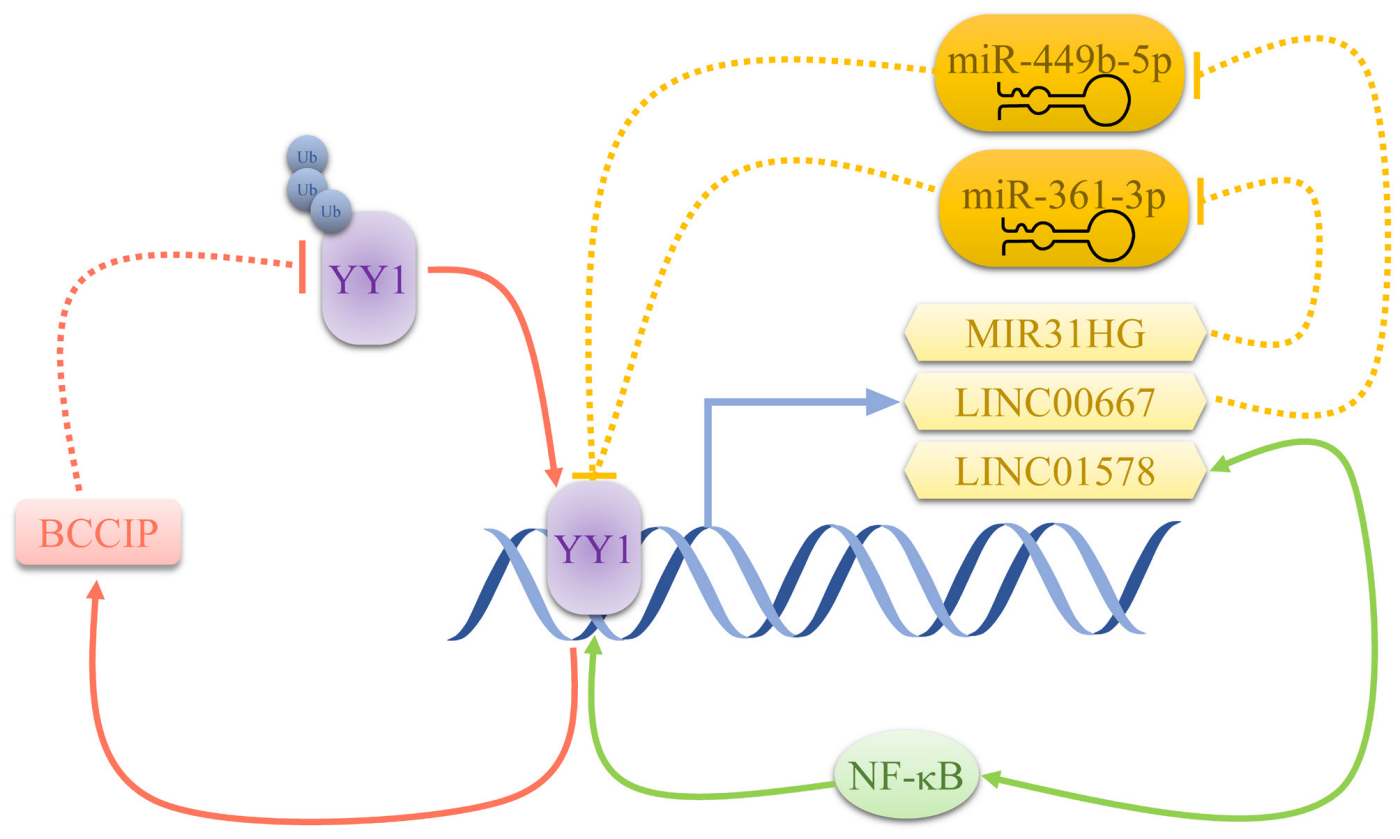
Schematic representation of YY1‐related feedback loops in previous studies of CRC. YY1 may transcriptionally activate some lncRNAs. In turn, these lncRNAs promote the expression and function of YY1 by either sponging the miRNAs that suppress YY1 expression or interacting with other transcriptional factors such as NF‐κB. YY1 may also promote the transcription of BCCIP who increases YY1 stability by inhibiting its ubiquitination.

Due to the interaction among YY1, lncRNAs and miRNAs detected in CRC cells, researchers attempted to probe the existence of YY1‐related feedback loop (Figure 1). Guo et al. found that LncRNA MIR31HG promotes YY1 expression on both mRNA and protein level via sponging miR‐361‐3p which negatively regulates YY1 expression. Without the suppression from miR‐361‐3p, overexpressed YY1 accelerates proliferation and glycolysis of CRC cells and the angiogenesis of human umbilical vein endothelial cells (HUVECs), meanwhile positively stimulating MIR31HG level in turn.^38^ The similar positive feedback loops were detected as YY1/LINC00667/miR‐449b‐5p axis^39^ and YY1/LINC01224/ miR‐485‐5p/myosins of class VI (MYO6) axis.^62^ Cooperating with NF‐κB, YY1 directly binds to the promoter of LINC01578 to enhance its activity. Conversely, LINC01578 activates NF‐κB signaling by directly binding the promoter of IκBβ encoding gene (NFKBIB) and recruiting EZH2 to NFKBIB promoter region to further repress NFKBIB expression.^63^

As mentioned above, BCCIP could bind directly to the residues 146–270 of YY1 and stabilize YY1 protein in HCT‐116 cell line presumably through the ubiquitin–proteasome‐mediated degradation pathway. The stably expressed YY1 in turn impacts the accessibility of BCCIP to DNA and improve the recruitment of YY1/BCCIP complex to the promoter region of itself, establishing the BCCIP‐YY1 feedback mechanism.^28^

## 5 INCONGRUENT OBSERVATIONS OF YY1 FUNCTION IN CRC

Considering CRC, the majority of studies demonstrate the oncogenic role of YY1, while several incongruences are reported (Table 2). For instance, loss of YY1 was reported to be significantly associated with aggressive phenotypes and prognosis in CRC patients.^13^, ^64^, ^65^ In research setting, YY1 plays a positive role of 5‐fluorouracil‐induced cytotoxicity in two CRC cell lines, HT‐29 and SW620. Further investigation showed that YY1 modulates the apoptotic response of both cell lines by indirectly interacting with BCL2L15, a member of Bcl‐2 family of apoptotic gene regulators.^13^ Future studies are desired to depict the nature of the postulated interaction.

## 6 CONCLUSION

**FIGURE 2:**
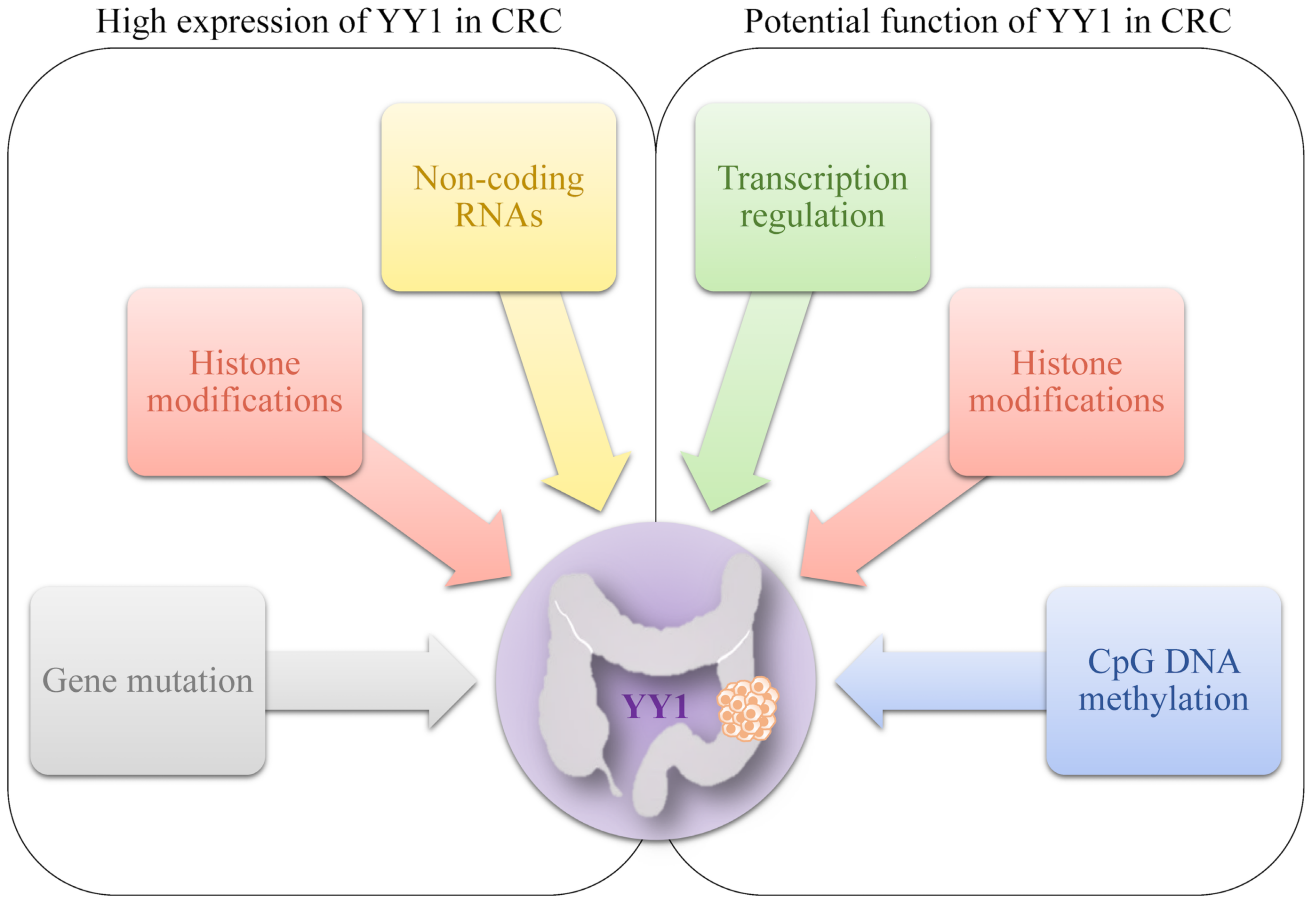
The illustration compasses the machineries that may attribute to the high expression of YY1 and the potential function of YY1 in CRC.

YY1, a puzzling and fascinating TF, hold an intrinsically disordered structure which ensures its multitasking ability but impedes us to view its full picture. The function of YY1 has been broadly studied in various disease models.^69^, ^70^, ^71^, ^72^, ^73^, ^74^, ^75^, ^76^ Generally speaking, YY1 plays a tumor promotion role in the majority of cancer types besides pancreatic cancer.^77^ In CRC, YY1 protein is highly expressed presumably stemming from impaired ubiquitination, promoter hypomethylation and negative regulation of ncRNAs.^26^, ^27^, ^29^, ^30^, ^31^, ^32^, ^33^, ^34^, ^35^, ^36^, ^37^, ^38^, ^39^, ^40^, ^41^, ^42^, ^43^, ^44^, ^56^ Highly expressed YY1 in CRC exert oncogenic function during the whole course of CRC. YY1 acts as a TF to suppress some classical anti‐cancer pathways (e.g., p53 signaling axis and Fas/FasL‐mediated apoptosis) but promote the oncogenic pathways (e.g., c‐myc signaling axis and Wnt signaling axis).^26^, ^27^, ^31^, ^32^, ^35^ Meanwhile, it may also involve the epigenetic modifications by cooperating with the histone repressive complex or other TFs. It is worth noting that the existence of feedback loop engaging YY1 and ncRNAs amplifies their joint oncogenic action (Figure 2).^28^, ^38^, ^39^, ^62^, ^63^ Whereas, controversial views are raised in terms of CRC treatment.^13^, ^64^, ^65^ It yet reminds us that the influence of YY1 on the evolution process of CRC and different therapeutic regimens might be overlooked in the past.

So far, there are sporadic clinical trials concerning YY1 as a prognostic predictor in breast cancer, follicular lymphoma, diffuse large B‐cell lymphoma, endotoxemia, and pediatric sepsis^70^, ^72^, ^73^, ^76^ (Table 3). Similar treatment management strategy might be further evaluated in CRC. Although inhibitors targeting YY1 is not currently developed, the applications of ncRNAs in cancer treatment are reported, some of which have entered a clinical trial stage.^78^, ^79^ Therefore, the targeted strategies that modulating the upstream ncRNAs of YY1 might have application prospects to suppress the oncogenic functions of YY1 in CRC. More detailed and elaborated studies are required to better understand the structure and function of YY1 which may ultimately help us to develop novel therapeutic strategies targeting YY1 or its interacting partners.

**TABLE 3:** Clinical trials concerning the expression and activity of YY1.

Disease	Study subjects	Interventions	Main findings	Clinical significance	Reference
Endotoxemia	Caucasian male volunteers	Different dose of intravenous LPS (endotoxin)	Leukocyte transcriptional signatures was changed, and the promoter regions of the LPS dose gene set were enriched for the binding sites of several transcription factors, including YY1.	Providing assist in assessing the severity of the insult in patients with abdominal sepsis.	^76^
Breast cancer	Breast cancer patients (*n* = 38)	Treatment arm: perioperative use of the β‐blocker (propranolol) and the COX2‐inhibitor (etodolac); Control arm: placebo	Transcriptome modulation analysis in PBMCs revealed treatment‐induced improvement in several transcription factors including YY1.	YY1 was used as an angiogenesis biomarker.	^73^
Pediatric sepsis	Children with sepsis (*n* = 30)	‐	Low level of YY1 in PBMCs was associated with poor prognosis.	YY1 expression in PBMCs may be used as a prognostic predictor.	^72^
FL and DLBCL	Archived human frozen lymph nodes (*n* = 106)	‐	High level of YY1 was associated with poor prognosis and rituximab resistance in both FL and DLBCL.	YY1 expression may be used as a prognostic predictor.	^70^
T1D	Recent‐onset T1D patients (*n* = 6) and healthy subjects (*n* = 5)	‐	In Treg cells, the risk single nucleotide polymorphism rs883868 disrupts YY1 binding and leads to the loss of enhancer–promoter looping mediated by YY1.	Explaining how noncoding variants affect the transcriptomes of two T‐cell subtypes that play critical roles in T1D pathogenesis.	^75^
NPC	244 NPC patients, 483 healthy subjects from endemic areas and 303 health subjects from non‐endemic areas	‐	Rp‐V1, a novel EBV subtype, impaired the transcription repression effect of YY1.	Partially explaining the elevated activation ability of EBV in NPC endemic areas.	^74^
Age‐associated skeletal muscle decline	Senior sportsmen (*n* = 15), healthy sedentary seniors (*n* = 9) and young participants (*n* = 5)	‐	YY1 was significantly upregulated in senior groups compared with young men group.	The upregulation of YY1 during aging might compensate for both mitochondrial dysfunction and insulin resistance.	^71^
HIV infection	HIV‐infected patients (*n* = 6)	IL‐2 and ART	The expression and activity of YY1 in PBMCs were down‐modulated during the treatment.	Providing a potential explanation of the transient raises in plasma viremia after receiving IL‐2 in the absence of ART.	^69^

Abbreviations: ART, antiretroviral therapy; DLBCL, diffuse large B‐cell lymphoma; EBV, Epstein–Barr Virus; FL, follicular lymphoma; HIV, human immunodeficiency virus; IL‐2, interleukin‐2; NPC, nasopharyngeal carcinoma; PBMCs, peripheral blood mononuclear cells; T1D, type 1 diabetes.

## AUTHOR CONTRIBUTIONS

**Zhiying Shao:** Writing – original draft (lead); writing – review and editing (equal). **Wendong Yang:** Writing – original draft (equal); writing – review and editing (lead). **Xiannan Meng:** Writing – original draft (equal); writing – review and editing (supporting). **Minle Li:** Writing – review and editing (equal). **Pingfu Hou:** Writing – review and editing (equal). **Zhongwei Li:** Writing – review and editing (equal). **Sufang Chu:** Resources (equal). **Junian Zheng:** Supervision (equal). **Jin Bai:** Supervision (equal).

## FUNDING INFORMATION

No specific funding was disclosed.

## CONFLICT OF INTEREST STATEMENT

The authors declare no competing interests.

## Data Availability

Data sharing is not applicable to this article as no new data were created or analyzed in this study.
